# Business cycle and mortality in Spain

**DOI:** 10.1007/s10198-021-01336-7

**Published:** 2021-06-23

**Authors:** María Cervini-Plá, Judit Vall-Castelló

**Affiliations:** 1 Universitat Autònoma de Barcelona, Edifici B. Campus UAB, Bellaterra (Cerdanyola del Vallès), 08193 Barcelona, Spain; 2 EQUALITAS, Madrid, Spain; 3 Universitat de Barcelona, Barcelona, Spain; 4 CRES (UPF), Barcelona, Spain

**Keywords:** Mortality, Health, Recessions, Business cycle, E32, I10, I12, I14, J14, J16

## Abstract

In the last couple of decades, there has been a lot of interest on the impact of macroeconomic fluctuations on health and mortality rates. Many studies, for different countries, find that mortality is procyclical. However, studies examining the effects of more recent recessions are less conclusive, finding mortality to be less procyclical, or even countercyclical. In this paper, using data of Spanish provinces from 1999 to 2016, we investigate how this relationship works in the context of a country that is subject to extreme business cycle fluctuations. Furthermore, we analyze the impact of unemployment for different mortality causes and we explore differences by sex, age group and level of education. In general terms, we find mortality to be procyclical so that when the economy is in a recession, mortality falls. When exploring mortality causes, we show that deaths from cardiovascular disease, cancer, senility, transport accidents and homicides are procyclical. By sex, we find procyclicality for both men and women. By age, mortality is procyclical for all age groups; however, the causes of death that result in this procyclical behavior are specific to each age group. By educational level, suicide appears as a countercyclical cause for individuals with intermediate levels of education.

## Introduction

There is a widely interest among social science researches and policymakers in understanding the relationship between macroeconomic fluctuations and health, with a particular focus on mortality.

Aggregated time-series data by Brenner [1, 2] disclosed that deaths by cardiovascular disease, cirrhosis, suicide and homicide, and infant mortality rates decrease when economic conditions improve. Nevertheless, the effects of business cycle conditions on mortality have become more controversial since the work by Ruhm [15], where he showed that mortality increases in periods of economic growth. In his paper, Ruhm combined time-series with cross-sectional data to better control for omitted variable bias.

In the last couple of decades, many studies have attempted to replicate these findings with the aim of understanding the specific causes that could explain this relationship using multiple techniques and datasets. However, the results of this large literature show ambiguous conclusions: on the one hand, there are many papers supporting the idea that mortality rates decrease during temporary economic recessions [6, 12–14, 21, 23, 24] so that “recessions are good for your health”. On the other hand, fewer studies reveal the opposite finding pointing towards health improvements during booms as suggested by the first papers on the literature in the 1970’s [5, 20].

In 2008 Edwards [4] pointed towards the importance of the level of aggregation used for the analysis. He used both micro and macrolevel data and found that, when using aggregated data, the results show a procyclical relationship between mortality and business cycle fluctuations while at the individual level the results are more mixed depending on the subgroup of the population studied. Therefore, his findings highlight the fact that the level of analysis plays a crucial role in the sign of the relationship precisely because business cycle effects are heterogeneous across individuals. Therefore, the composition of the population plays an important role in defining the association at the macrolevel.

Another more recent paper that explore the opposite-direction effects of contextual and individual unemployment on mortality is Tapia Granados et al. [24]. They show how an increase in the unemployment rate correlates with a decrease in overall mortality or mortality due to most causes (procyclicality). However, at the same time, at the individual level, to suffer unemployment (because of being fired, plant closure or whatever) seems clearly connected with an increase in the risk of death. Using US data they find that compared to be employed, for those who experienced unemployment the hazard of death was increased by an amount equivalent to 10 extra years of age, and, at the same time, each percentage-point increase in the state unemployment rate (contextual unemployment) reduced the mortality hazard in all individuals by an amount equivalent to a reduction of 1 year of age. In line with this result at the microlevel, Sullivan and von Wachter [19] find that the risk of death is higher in individuals exposed to unemployment.

The recent recession that affected the global economy in 2008 marked a new opportunity to study this relationship. The new wave of papers looking at the relationship between mortality and business cycle conditions find even more diverging conclusions: some authors point towards a reduction in the previously documented procyclicality when using post-2008 data as compared to papers using pre-2008 data [7, 8, 13, 14, 17] another group of authors find the relationship to be countercyclical [9, 11] while still a third group of authors report mortality to be unrelated to macroeconomic conditions [16].

Ruhm [16] suggests that changes in health behaviors such as the reduction in drinking, obesity, smoking and physical inactivity during recessions represent a credible and powerful mechanism that backs up the procyclicality argument. Cutler et al. [3] find that pollution also has explanatory power in the relationship between mortality and business cycle conditions as they report that, in places where pollution is low (i.e. agricultural economies), mortality rates are countercyclical. However, when pollution is important and is highly determined by the level of economic activity, mortality rates become procyclical. This finding is consistent with the work of Ionides et al. [10] showing that procyclical mortality is driven mostly by respiratory diseases and traffic injuries. Similarly, Strumpf et al. [18] estimate that increases in the local unemployment rates are associated with reductions in all-cause mortality rates and, 60% of this decrease, is explained by cardiovascular diseases. They also find reductions in motor vehicle fatalities during economic recessions, especially for men under age 65. All these new papers strongly suggest the need to explore the contribution of each specific cause of death in order to fully understand the relationship between mortality and business cycle conditions.

Nonetheless, the factors explaining the divergence of findings in the literature are not entirely clear yet. Some authors have provided some examples: Ruhm [16] suggests that changes over time in the relationship between business cycle conditions and mortality may be driven by the fact that economic instability over time is poorly measured when using short periods of analysis. Another possible reason is the fact that the mechanisms behind the association between macroeconomic conditions and mortality may not be stable over time due to the changes in institutions or changes in health behaviors. Cutler et al. [3] also point in this direction, since they find that the intensity and even the direction of the relationship between mortality rates and macroeconomic conditions can be directly related to government spending levels. Going one step further, Stevens et al. [17] presents evidence that staff in nursing homes in the USA has a counter-cyclical behavior so that (at least part) of the mortality increases during booms can be explained by fluctuations in the quality of the health care provided.

**Fig. 1:**
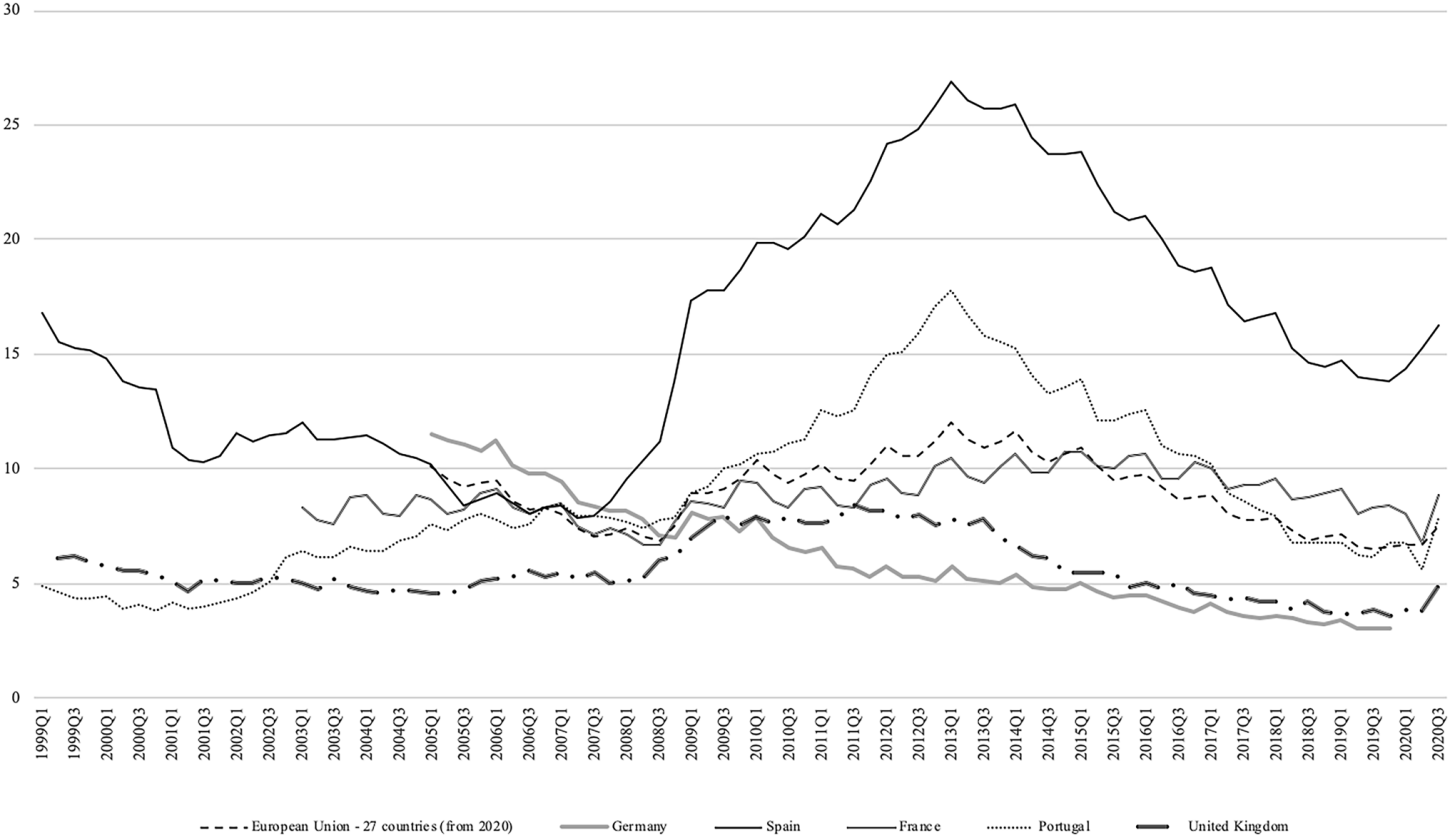
Unemployment rates (1999–2013). Eurostat

Our paper contributes to this literature by examining the relationship between macroeconomic conditions and mortality in Spain, a country that has been severely hit by the recent economic crisis. Using very rich administrative data (mortality registers) covering the period 1999–2016, we link local (provincial) level unemployment rates to local level mortality rates while controlling for the age structure of the population, regional and time fixed effects, as well as regional trends. The strong variability in business cycle conditions over time in Spain (as seen in Fig. 1) represents a powerful element that strengthens the identification strategy and potentially allows us to increase the precision of the estimates. We believe that strong variations in the main explanatory variable in Spain vis-à-vis the relatively smaller variation in other developed countries previously studied in the literature is a comparative advantage that will help us better identify the relationship between business cycle conditions and mortality rates. We also explore the impacts for specific causes of death as well as differences by age, sex and educational level.

As far as we are aware, the Spanish case has been previously studied in Tapia Granados [22] and Regidor et al. [13, 14]. The paper by Tapia Granados [22] explores the relationship between economic conditions and mortality for Spain using data from 1980 to 1997. His results show a procyclical relationship for the Spanish case although the size of the effects are smaller when compared to other countries: more specifically, he estimates a drop of 0.11% in mortality for a one percentage point increase in the local unemployment rate. Our paper presents several novelties with respect to Tapia Granados [22]. First, we include observations until 2016 which allows us to capture the impacts of the recent economic crisis, which represented an unprecedented shock for the Spanish economy that raised unemployment rates over 25%, as well as periods with an extremely booming economy (such as 2005–2007) with unemployment rates reaching minimum historical values below 10%. Therefore, the effects that we find are much larger than those reported in Tapia Granados [22] as our results show that mortality reduces by 0.42% for a once percentage point increase in the local unemployment rate. Furthermore, having this updated information is also an important contribution to the recent literature as several papers have reported potential changes in the relationship between business cycle conditions and mortality in recent years. Our results show that, for the Spanish case, mortality is still strongly procyclical and this relationship has not changed in the recent years, as has been documented for other countries. On the contrary, the relationship has become larger as the coefficient in our estimates is larger than that reported in previous papers for Spain. Furthermore, we present a detailed analysis of the impact for several sources of death which allows us to better understand the mechanisms driving this relationship. Finally, we also focus on sex, age and educational differences that were previously undocumented.

More recently, we find two papers Regidor et al. [13, 14] that analyse the mortality rate in Spain before and during the Great Recession, using Census data and following individuals until 2011. Regidor et al. [14] analyse mortality trends in different socioeconomic groups, quantifying the change within each group. They classified individuals by socioeconomic status (low, medium, or high) using two indicators of household wealth: household floor space and number of cars owned by the residents of the household. They find that in all socioeconomic groups, the all-cause mortality rate declined more during the first 4 years of the crisis period (2008–11) than in the 4 years receding the crisis (2004–07), except for women in the high socioeconomic group, whose mortality declined less during the crisis. Exploring cause-specific mortality rates, they show a similar pattern, except for cancer in all women and for traffic and other unintentional injuries in women in the high socioeconomic group. Furthermore, the acceleration of the downward linear trend in mortality was higher in the lowest socioeconomic group than in the highest group for all-cause deaths and for most of the specific causes of death analysed. Regidor et al. [13] analyse mortality rates for employed, unemployed and inactive individuals. They find that for employed and unemployed men, mortality rates increased until 2007 and then declined, whereas in employed and unemployed women, mortality rates increased and then stabilized during 2008–2011. The mortality rate among inactive men and women decreased throughout the follow-up. Thus, they find that during the economic crisis, the upward trend in the rate of mortality from all diseases was inverted in the groups of employed and unemployed subjects at baseline. Compared with these two interesting most recent papers, our article provides a longer period, a record of all the people who have died in Spain and a detailed analysis by age and educational level.

Our results suggest that, when the economy is in a recession, mortality rate falls. This result is consistent with the findings in Tapia Granados [22] and suggests that the relationship between business cycle conditions and mortality has not changed in the last 30 years. Additionally, our study detects that, while cardiovascular deaths, senility diseases, cancer and deaths by transport accidents are procyclical, countercyclical patterns emerge for suicide and diabetes. With respect to sex differences, our results show that men are significantly more affected by transport accidents than women in Spain. Although mortality is procyclical for all age groups , the causes that lead this pro-cyclicality are different in each age segment. Finally, suicide appears as a countercyclical cause for intermediate education levels.

This paper is organized as follows. "Methodology" section presents our methodology. "Data and descriptive statistics" section explains the dataset used and our sample selection. "Results" section shows the main results and "Heterogeneous effects" section explores heterogeneities by sex, age and education. Finally, "Conclusion" section concludes.

## Methodology

To analyse the relationship between macroeconomic conditions and mortality rates, we follow very close Ruhm [16]. Our dependent variable is the mortality rate calculated as the number of deaths in a particular cell (we use different definitions of a cell) divided by the population in that particular cell. More specifically, we estimate the following equation:

1 $$\begin{aligned} ln(M_{kjt})= & {} \alpha _{kj}+U_{kjt}\gamma +\lambda _{kt}+H_{j}*t+A15_{jt}\eta \nonumber \\&+A65_{jt}\delta +\epsilon _{kjt} \end{aligned}$$

where $M_{kjt}$ is the mortality rate from diseases *k* in province *j* at certain moment of time *t*, *U* is the unemployment rate in that province at that time (a proxy of the economic conditions), $\alpha$ are province fixed-effects that control for time-invariant province characteristics (such as persistent lifestyle disparities between residents of two different provinces), $\lambda$ accounts for calendar time fixed effects (which capture any determinant of mortality that vary over time but in the same way across provinces: e.g. advances in widely used medical technologies or behavioural norms), $H_{j}*t$ is a province-specific linear trend and $\epsilon$ is the error term. Therefore, $\gamma$ is our parameter of interest and captures the effects of the cycle on mortality. Finally, we acknowledge that the age structure is an important determinant of total mortality and mortality due to specific causes. Thus, to control for the demographic structure of the population and any potential changes over time (that might not be fully captured by the province-specific linear trends), we also include as demographic controls the proportion of the population above 65 years old $A65_{jt}$ as well as the proportion of the population below 15 years old $A15_{jt}$. In that way, the results of the model reflect how age-adjusted mortality responds to the changes in economic conditions.

To estimate the relative effects we can estimate, following the same idea than Ruhm [16]:

$$\begin{aligned} (e^{\hat{\gamma } k}-1)*100\% \end{aligned}$$

This expression gives us the predicted percentage change in mortality from source *k* resulting from a one percentage point increase in the unemployment rate. These relative effects are translated into absolute numbers through estimates of:

$$\begin{aligned} (e\Delta ^{\hat{\gamma } k}-1) *\pi _{k}D, \end{aligned}$$

where $\Delta ^{\hat{\gamma } k}=\hat{\gamma }_{2k}-\hat{\gamma }_{1k}$ and $\pi _{k}$ is the share of deaths due to source *k* and *D* is the average annual number of deaths (384,710).

In particular, we estimate Eq (1) for the total mortality rate and by specific mortality causes using annual data by province. Furthermore, we explore differences in the impact of business cycle conditions on annual mortality rates by sex, age groups and educational level using group-specific outcomes.

We consider 52 geographic units that, for simplicity, we call provinces. However, strictly speaking what we include corresponds to 50 provinces plus Ceuta and Melilla, that are autonomous cities. All regressions are weighted by the squared root of the population in each geographic unit and time, and standard errors are clustered by geographic unit.

## Data and descriptive statistics

As an indicator of macroeconomic conditions, we use the unemployment rate. We obtain annual and quarterly unemployment rate information for each province from the National Institute of Statistics in Spain (INE).

We use mortality registers for the period 1999–2016 also from the INE. The restricted access version of the files allow us to include information on the type of diseases that caused the death. The registers are collected at the individual level and include all deaths in Spain from 1990 to 2016, the diseases that caused the death and some individual characteristics such as sex, age, education and province of the person that died. We collapse the individual information by province for each year to get the number of individuals dying in each province and time period. We aggregate the information for total mortality but also for different causes of death. To do the analysis of cause-specific mortality we classify the different mortality causes in 12 categories: cardiovascular, cancer, respiratory, senility, Alzheimer, diabetes, digestive, other diseases, transport accidents, other accidents, suicides and homicides. To calculate mortality rates, we divide the number of deaths by the population (and multiply it by 1000), using information on population numbers in the same province and year.

In addition to total annual mortality rates for each province, we construct sex-specific death rates, mortality rates for five age groups (< 25, 25–44, 45–64, 65–74, > 74), as well as by level of education for those aged over 25 years old (we only have information about education from 2012 onwards).

**Fig. 2:**
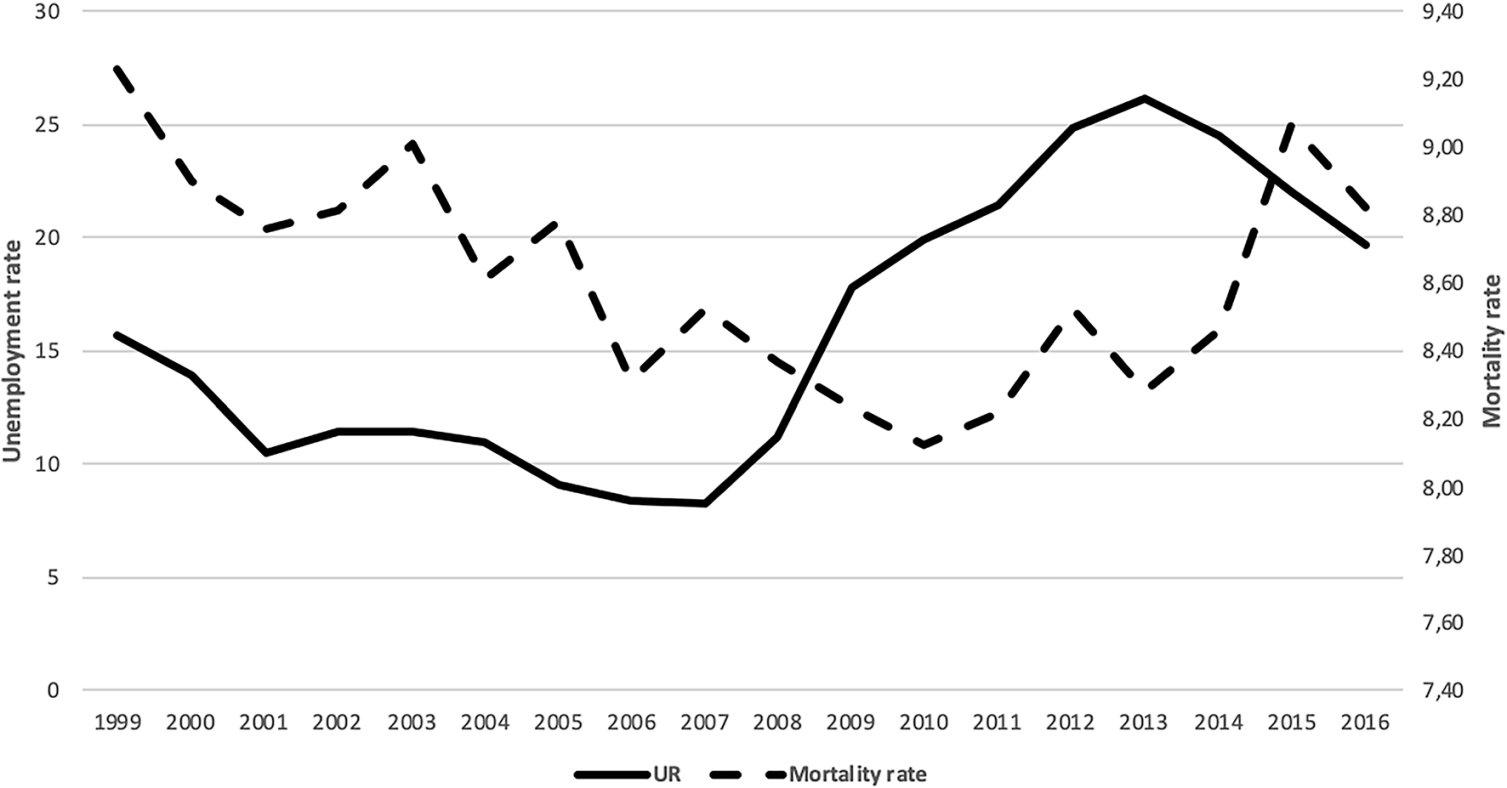
Mortality rate and unemployment rate

Table 5 in Appendix presents information, for each of the years included in our database, on the total number of people dying (total mortality), the total number of individuals (population) and the mortality rate. As can be seen in the table, both the number of people who die and the total population is increasing in our period. However, the mortality rate has more volatility and it is increasing in some periods and decreasing in others. Figure 2 shows the evolution of the mortality rate and the unemployment rate in Spain from 1999 to 2016. The relationship between the two variables is not clear; while in the first period it seems that both variables move in parallel, after the crisis it looks like they go in opposite directions, recovering a the parallel path in the last years of the analyzed period.

Table 6 in Appendix shows some descriptive statistics of our sample. With respect to the causes of death, we can observe that the most common causes of death are: cardiovascular problems, cancer and respiratory problems. As for age, we can see that the proportion of individuals dying is highly concentrated in the oldest age group. More than 70% of the deaths occur for people who have more than 75 years old. Regarding the level of education, most of individuals in our sample have a relatively low level of education. This is due to the fact that the education level of the elderly is much lower than the average for the entire population.

**Fig. 3:**
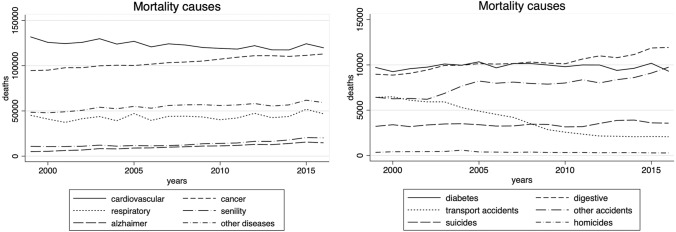
Evolution mortality causes

Figure 3 in the Appendix shows the evolution of the different mortality causes in the period analyzed. On the left panel we can see a slight reduction in deaths from cardiovascular problems, an increase in cancer mortality and diseases associated with an aging population such as senility and Alzheimer. On the right hand side of the panel, we can see a continuous reduction in traffic accidents throughout the period, which coincides with an increased governmental effort to raise public awareness on the importance of enforcing traffic rules. Other types of accidents are increasing during the period and suicides appear to have increased after the crisis.

## Results

In the first row of Table 1, we examine the relationship between economic conditions and total mortality rates with annual information. Our parameter of interest, the local level unemployment rate, has a significant and negative coefficient. Thus, we find a procyclical relationship between business cycle conditions and mortality in Spain during our sample period. That is, once long-term trends are adjusted for, when the local unemployment rate increases because the economy is in a recession, mortality falls. We find that a one percentage point increase in the local unemployment rate reduces the mortality rate by 0.42%. The estimation include year and province fixed effect, linear trends, and age controls.

**Table 1:** Effect of a one percentage point increase in the unemployment rate on the mortality rate due to all causes

Mortality rate	Effect	Standard error
All causes	− 0.0042***	(0.0009)
Cardiovascular	− 0.0072***	(0.0017)
Cancer	− 0.0022***	(0.0008)
Respiratory	− 0.0025*	(0.0015)
Senility	− 0.0146***	(0.0036)
Alzheimer	0.0001	(0.0023)
Diabetes	0.0006	(0.0061)
Digestive	− 0.0039	(0.0027)
Other diseases	− 0.0021	(0.0018)
Transport accidents	− 0.0147***	(0.0039)
Other accidents	− 0.0088*	(0.0044)
Suicide	− 0.0035	(0.0025)
Homicide	− 0.0239**	(0.0110)
Men	− 0.0041***	(0.0008)
Women	− 0.0025***	(0.0007)

Number of observations: 936. All estimations include Year FE, Province FE, Linear trends and demographic controls

Robust standard errors in parentheses, *** p < 0.01, ** p < 0.05, * p < 0.1

In the following rows of Table 1, we present separate results for each of the 12 categories of cause-specific mortality. We find that some causes are strongly and significantly procyclical, such as cardiovascular diseases, cancer, senility diseases, transport accidents and homicide. Another group of diseases have a negative sign but the coefficient is only significant at the 10% level, such as respiratory diseases or other accidents. We also find that a number of diseases show a counter-cyclical relationship with business cycle conditions, but not significant, such as Alzheimer and diabetes. Some of these relationships have been found in papers looking at the same question in other countries [15, 16]. Therefore, some of the sources of deaths seem to be procyclical across several countries and this is particularly the case for transport accidents and cardiovascular diseases. At the macrolevel, our data (and the majority of the previous literature) seems to support the idea that in times of economic booms, and higher employment probabilities, there are more cardiovascular deaths. As we mentioned in the introduction, Tapia Granados et al. [24] explore the paradox between what is found at the micro and macroeconomic levels. Although it is true that higher unemployment at the individual level can generate more mortality associated with greater stress, at the macrolevel, a higher unemployment rate is associated (in most countries) with lower mortality from most causes (including cardiovascular). For example, the reduction in mobility leads to a lower number of transport accidents.

## Heterogeneous effects

In the last two rows of Table 1, we present results separately for men and women using annual data. We find a statistically significant procyclical coefficient for both, men and women. For the case of men, we estimate that a one percentage point increase in unemployment leads to a reduction in mortality by 0,41%. For women the coefficient is also negative but somewhat smaller; a one percentage point increase in the unemployment rate is associated with a reduction of 0.25% in mortality.

Table 7 in the Appendix presents the results by sex for the different causes. For men, we find that the coefficients that appear most significant are cardivascular, cancer, senility and transport accidents. These coefficients have a negative sign, which shows a procyclical relationship. When unemployment increases in a recession, deaths due to cardiovascular problems decrease partly because individuals are exposed to less job-related stress and eating habits improved due to more home cooked meals. At the same time, as mobility is reduced during unemployment spells, there are lower deaths due to transport accidents. For the case of women, we observe the same pattern but the estimated coefficients are somewhat smaller. Some causes are only significant either for men or for women. For example, we find that the coefficient of homicides and other accidents is significant and negative (showing procyclicality) for men. While in the case of women we find procyclicality in deaths from digestive diseases.

A second exploration exercise that we are interested in this article is to analyse the differences in the cyclicality of mortality by age group. As we explained in the descriptive section, we have five age groups (< 25, 25–44, 45–64, 65–74, > 74). In Table 2 we can see that, for all age groups, we find strong evidence that mortality is procyclical and significant. That means that, when unemployment increases, mortality decreases and vice versa. However, there are important differences in the causes that lead this procyclicality, which vary for each age group.

In Table 8 in the Appendix, we explore the results by age group and for different causes of deaths. There is considerable heterogeneity across different sources of deaths. For those under the age of 25 (which we can observe in more detail in the first column of the table), there are three coefficients that have a negative and significant sign (evidence of prociclicality): deaths due to cardiovascular diseases, other diseases and transport accidents. However, it is important to mention that cardiovascular diseases are not important in this age group. In the age group 25–44, as we can observe in the second column, we do not find any significant coefficient. In the third column we show the results for individuals aged 45–64 and we find a negative coefficient in the following mortality causes: cardiovascular, respiratory diseases, diabetes, other diseases and transport accidents. For the age group 65–74 (column 4), we can see a negative coefficient in deaths due to cardiovascular problems and respiratory diseases. Finally, we present detailed results for those over 74 and we obtain a significant and negative coefficient for: cardiovascular problems, digestive and other diseases. Therefore, although mortality is procyclical for all age groups, the causes that lead this procyclicality are somewhat different for the different age segments.

**Table 2:** Effects on age-specific mortality rates associated with a one-percentage point increase in the unemployment rate

Mortality rate	0–25	25–44	45–64	65–74	> 74
Unemployment	− 0.0079** (0.0026)	− 0.0024** (0.0011)	− 0.0032** (0.0015)	− 0.0042*** (0.0011)	− 0.0029*** (0.0010)
Observations	936	936	936	936	936
R-squared	0.8773	0.8618	0.9889	0.9960	0.9994

All estimations include Year FE, Province FE, Linear trends

Robust standard errors in parentheses, *** p < 0.01, ** p < 0.05, * p < 0.1

A third heterogeneity exercise that we implement distinguishes across different levels of education. We classify individuals according to the highest level of education that they have obtained. As we only want to include those individuals that have already completed their desired level of education, we restrict the sample to those aged over 25 years old. Unfortunately we only have this information from 2012 onwards. In our dataset, we can distinguish between five education levels: less than primary school, primary school, secondary, high school and university. As we can see in Table 3, we do not obtain any significant coefficient for the regressions of all cause mortality by different educational groups. However, when analyzed by cause, in Table 9 in the Appendix, we observe that suicides show a countercyclical pattern, as they increase during crises periods and decrease in moments of expansion, for individuals with an intermediate level of education.

**Table 3:** Effects on the death rate of groups of different educational level associated with a one-percentage point increase in the unemployment rate

Mortality rate	Less primary	Primary	Secondary	High school	University
Unemployment	0.0007 (0.0021)	0.0023 (0.0014)	0.0001 (0.0016)	0.0021 (0.0029)	0.0034 (0.0026)
Observations	260	260	260	260	260
R-squared	0.9939	0.9960	0.9966	0.9633	0.9716

All estimations include Year FE, Province FE, Linear trends and demographic controls

Robust standard errors in parentheses, *** p < 0.01, ** p < 0.05, * p < 0.1

Finally, an important exercise is to calculate how much these coefficients represent in terms of the number of deaths. Table 4 shows that a one percentage point increase in the unemployment rate decreases the total number of deaths by, on average, 1612 per year (with a 95 % confidence interval ranging from 936 deaths to 2288). By sex, the prediction of the effect of a 1 point increase in the unemployment rate for men is higher (801 fewer deaths) than for women (472 fewer deaths). By causes, the main cause that decreases in a recession is cardiovascular deaths; a 1 point increase in the unemployment rate decreases cardiovascular deaths by 805 per year. Finally, we can also predict that a 1 point increase in the unemployment rate decreases cancer death by 232, transport accidents death by 28 and homicides by 9 per year.

**Table 4:** Predicted number of deaths

Population sub-group	Number of deaths	95%Conf. interval
All death	− 1612***	− 936, − 2288
Males	− 801***	− 495, − 1107
Females	− 472***	− 213, − 730
Cardiovascular for all	− 805***	− 434, − 1176
Cancer	− 232***	− 67, − 398
Transport accidents	− 28***	− 14, − 43
Homicide	− 9**	− 1, − 17

To calculate the predicted number of death we use $(e^{\hat{\gamma } k}-1)\cdot {\pi _{k}D}$ where $\pi _{k}$ is the share of deaths

due to source k and *D* is the average annual number of deaths (384,710)

95%confidence intervals are estimated as $(e^{\hat{\gamma } k\pm 1.96\cdot {s_{k}}}-1)x \pi _{k}D$ for $s_{k}$ standard error

*** p<0.01, ** p<0.05

## Conclusion

This paper shows that, during the period 1999–2016, mortality exhibits a procyclical pattern in Spain; that is, when the local unemployment rate increases mortality falls. Exploring the impact of unemployment for different mortality causes we show that deaths from cardiovascular disease, cancer, senility and transport accidents are strongly procyclical. However, countercyclical patterns have emerged for diabetes and suicide.

When we study the effects by sex, we see that, for both men and women the coefficient is negative (procyclicality) although the effects for men are somewhat higher. Again, cardiavascular, senility and transport accident causes seem to lead that procyclicality. Mortality is procyclical for all the age groups analysed; however, the causes that foster this procyclicality are slightly different in each age group. For example, for those between 45 and 65 respiratory problems and transport accidents explain the procyclicality pattern. On the other hand, procyclicity of deaths due to cardiovascular problems appear in a significant manner in all age groups (except those of 25–45). By educational level, suicide appears as a countercyclical cause (increasing in recessions and decreasing in the growing seasons) for individuals with intermediate education levels.

We believe that our paper provides new evidence on the relationship between business cycle conditions and mortality with respect to the previous literature. More specifically, our data allows us to study this relationship for a more recent period and for a country that has been exposed to an extremely strong recession in the mid-2008. With respect to the article of Tapia Granados [22], we are able to explore a more recent period, which allows us to capture the impacts of the recent economic crisis. As our results are consistent with his findings, we are confident when concluding that the sign and significance levels of the relationship between business cycle conditions and mortality in Spain has not changed in the last 30 years. However, we report that the effect of the business cycle on mortality has become larger in more recent years, as the coefficient in our estimates is larger than that reported in the previous papers for Spain.

Compared with Regidor et al. [13, 14] our article uses administrative data derived from the mortality registers, which is available for every year included in our study and, therefore, allows us to include a longer period of time than Regidor et al. [13, 14], which use Census data. Furthermore, we are able to present a detailed analysis of the impact for several sources of death, which allows us to better understand the mechanisms driving this relationship, as well as a detailed analysis by sex, age and education, that was previously undocumented.

## Appendix

See Appendix Table 5, Fig. 3, and Tables 6, 7, 8, 9.

**Table 5:** Total number of deaths, population and mortality rate (per thousand population)

Year	Deaths	Population	Mortality rate
1999	371,102	40,202,160	9.23
2000	360,391	40,499,791	8.90
2001	360,131	41,116,842	8.76
2002	368,618	41,837,894	8.81
2003	384,828	42,717,064	9.01
2004	371,934	43,197,684	8.61
2005	387,355	44,108,530	8.78
2006	371,478	44,708,964	8.31
2007	385,361	45,200,737	8.53
2008	386,324	46,157,822	8.37
2009	384,933	46,745,807	8.23
2010	382,047	47,021,031	8.13
2011	387,911	47,190,493	8.22
2012	402,950	47,265,321	8.53
2013	390,419	47,129,783	8.28
2014	395,830	46,771,341	8.46
2015	422,568	46,624,382	9.06
2016	410,611	46,557,008	8.82

**Table 6:** Proportions of deaths in total mortality by cause of death or characteristic of the deceased Source: Spanish mortality registers 1999–2016, Spanish National Institute of Statistics

Characteristic	Percentage
Sex	
Men	50.9
Women	49.1
Causes	
Cardiovascular	29.2
Cancer	27.5
Respiratory	11.4
Senility	4.9
Alzheimer	3.6
Diabetes	2.3
Digestive	2.9
Other diseases	14.4
Transport accidents	0.5
Other accidents	2.4
Suicides	0.9
Homicides	0.1
Age	
< 25	0.6
25–44	1.8
45–64	11.5
65–74	13.3
> 74	72.8
Education	
Less than primary school	27.8
Completed primary school	34.9
Compulsory secondary education (ESO)	19.9
High school and vocational education	8.1
University degree	9.3

**Table 7:** Effect on different causes by sex

Diseases	Men	Women
Cardiovascular	− 0.0066***	− 0.0050***
Cancer	− 0.0019**	− 0.0014**
Respiratory	− 0.0024	− 0.0004
Senility	− 0.0148***	− 0.0101***
Alzheimer	− 0.0013	0.0003
Diabetes	− 0.0002	0.0026
Digestive	− 0.0023	− 0.0042**
Other diseases	− 0.0038**	− 0.0009
Transport accidents	− 0.0188***	− 0.0145***
Other accidents	− 0.0080**	0.0020
Suicide	− 0.0042	0.0053
Homicide	− 0.0352**	− 0.0217

Number of observations: 936 for each estimation. All estimations include Year FE, Province FE, Linear trends and demographic controls

Robust standard errors in parentheses, **** p* < 0.01, *** p* < 0.05, ** p* < 0.1

**Table 8:** Different cause by age group

Diseases	0–25	25–44	45–64	65–74	> 74
Cardiovascular	− 0.0288*	− 0.0035	− 0.0079***	− 0.0090***	− 0.0067***
Cancer	0.0040	0.0014	0.0025	0.0010	0.0003
Respiratory	− 0.0027	− 0.0049	− 0.0067**	− 0.0043*	− 0.0008
Senility	− 0.0045	0.0043	0.0113	− 0.0024	− 0.0016
Alzheimer	0.0000	− 0.0136	0.0184	− 0.0051	0.0020
Diabetes	− 0.0039	0.0259	− 0.0109*	− 0.0047	− 0.0011
Digestive	− 0.0257	0.0140	− 0.0009	− 0.0002	− 0.0039*
Other diseases	− 0.0100**	− 0.0033	− 0.0082***	− 0.0049**	− 0.0031**
Transp Acc.	− 0.0142**	0.0059	− 0.0143***	− 0.0093	− 0.0098*
Other Acc.	− 0.0113	− 0.0010	− 0.0063	0.0027	0.0064
Suicide	− 0.0019	− 0.0005	− 0.0100**	− 0.0054	− 0.0073*
Homicide	− 0.0102	− 0.0088	− 0.0141	− 0.0077	0.0074

Number of observations: 936 for each estimation. All estimations include Year FE, Province FE, Linear trends

Robust standard errors in parentheses, **** p* < 0.01, *** p* < 0.05, ** p* < 0.1

**Table 9:** Different cause by education

Diseases	Less primary	Primary	Secondary	High school	University
Cardiovascular	0.0010	0.0030	− 0.0004	− 0.0007	0.0085
Cancer	− 0.0040	0.0015	− 0.0042	0.0081*	− 0.0015
Respiratory	0.0052	0.0021	− 0.0030	− 0.0049	− 0.0003
Senility	0.0137	0.0046	0.0030	0.0135	− 0.0186
Alzheimer	0.0033	− 0.0052	0.0145	0.0193	0.0134
Diabetes	− 0.0004	0.0164	0.0217	− 0.0161	0.0403
Digestive	0.0055	0.0088	0.0111	− 0.0406	0.0109
Other diseases	− 0.0016	− 0.0027	0.0031	− 0.0009	0.0021
Transp Acc.	− 0.0237	0.0209	0.0043	− 0.0099	− 0.1208*
Other Acc.	− 0.0078	0.0066	0.0261	0.0013	− 0.0012
Suicide	0.0059	0.0345	0.0175	0.0779**	0.0146
Homicide	− 0.1023	0.0564	0.1237	0.1857	0.0254

Number of observations: 936 for each estimation. All estimations include Year FE, Province FE, Linear trends and demographic controls

Robust standard errors in parentheses, **** p* < 0.01, *** p* < 0.05, ** p* < 0.1
